# Herpes simplex virus-1 triggered hemophagocytic lymphohistiocytosis in a patient with granulomatosis with polyangiitis

**DOI:** 10.4322/acr.2021.395

**Published:** 2022-08-17

**Authors:** Vanessa A. States, Meghan E. Kapp

**Affiliations:** 1 Vanderbilt University Medical Center, Department of Pathology, Microbiology & Immunology, Nashville, TN, USA

**Keywords:** Herpes simplex, Lymphocytes, Lymphohistiocytosis, Hemophagocytic, Macrophages, Vasculitis, Central Nervous System

## Abstract

Hemophagocytic lymphohistiocytosis (HLH) is a rare, aggressive hyperinflammatory syndrome in which an inciting event triggers massive, uninhibited activation of T lymphocytes and macrophages. Although viral infections are the most common trigger of HLH, cases of HSV-1 induced HLH are rare in adults. We present the case and postmortem findings of a 27-year-old woman diagnosed with HLH in the setting of immunosuppression for the treatment of granulomatosis with polyangiitis (GPA). Autopsy revealed evidence of herpes simplex virus-1 (HSV-1) infection and no findings suggestive of GPA recurrence.

## CASE REPORT

A 27-year-old woman presented to the emergency department with a four-day history of nausea, vomiting, diarrhea, and high fevers. Her medical history was significant for pituitary granulomatosis with polyangiitis (GPA) status post pituitary resection with ventriculoperitoneal shunt and treated with long term immunosuppressive therapy (azathioprine) and high dose steroids for a recent recurrence. She additionally had hypertension, diabetes, and polycystic ovarian syndrome. Physical examination and initial laboratory studies were significant for fever (39.6° C), tachycardia (162 bpm), leukopenia (WBC 3.3 x 10^9^/L - reference value [RV] 4.5-11.0 x 10^9^/L), thrombocytopenia (platelets 126 x 10^9^/L; RV 150-400 x 10^9^/L), mild transaminitis (AST 190 U/L - RV 0-35 U/L; ALT 55 U/L - RV 7-56 U/L; ALP 85 U/L - RV 44-147 U/L), and normal renal function (serum creatinine [SCr] 1 mg/dL - RV 0.59 - 1.04 mg/dL; BUN 11 mg/dL - RV 5.88 - 23.8 mg/dL). Blood and urine cultures were collected and the patient was started on empiric broad-spectrum antibiotics. Infectious Disease and Rheumatology were consulted for broader infectious workup and to investigate the possibility of GPA recurrence, respectively.

The patient continued to experience intermittent fevers and abdominal pain. An abdominal ultrasound revealed hepatosplenomegaly, which was not appreciated on physical examination. On hospital days four and five, her transaminitis (AST 1,661 U/L, ALT 238 U/L, ALP 91 U/L), and pancytopenia (WBC 0.9 x 10^9^/L, RBC 3.21 x 10^9^/L - RV 3.5-5.5 x 10^12^/L; platelets 69 x 10^9^/L) worsened, and she developed acute kidney injury (SCr 2.34 mg/dL, BUN 17.92 mg/dL), hyperferritinemia (30,987 µg/L - RV 20-220 µg/L), and hemodynamic instability. Clinical suspicion for hemophagocytic lymphohistiocytosis (HLH) prompted Hematology involvement. A bone marrow biopsy was obtained and high dose Methylprednisolone (1,000 mg IV infused over 30 minutes every 24 hours) was initiated.

**Figure 1:**
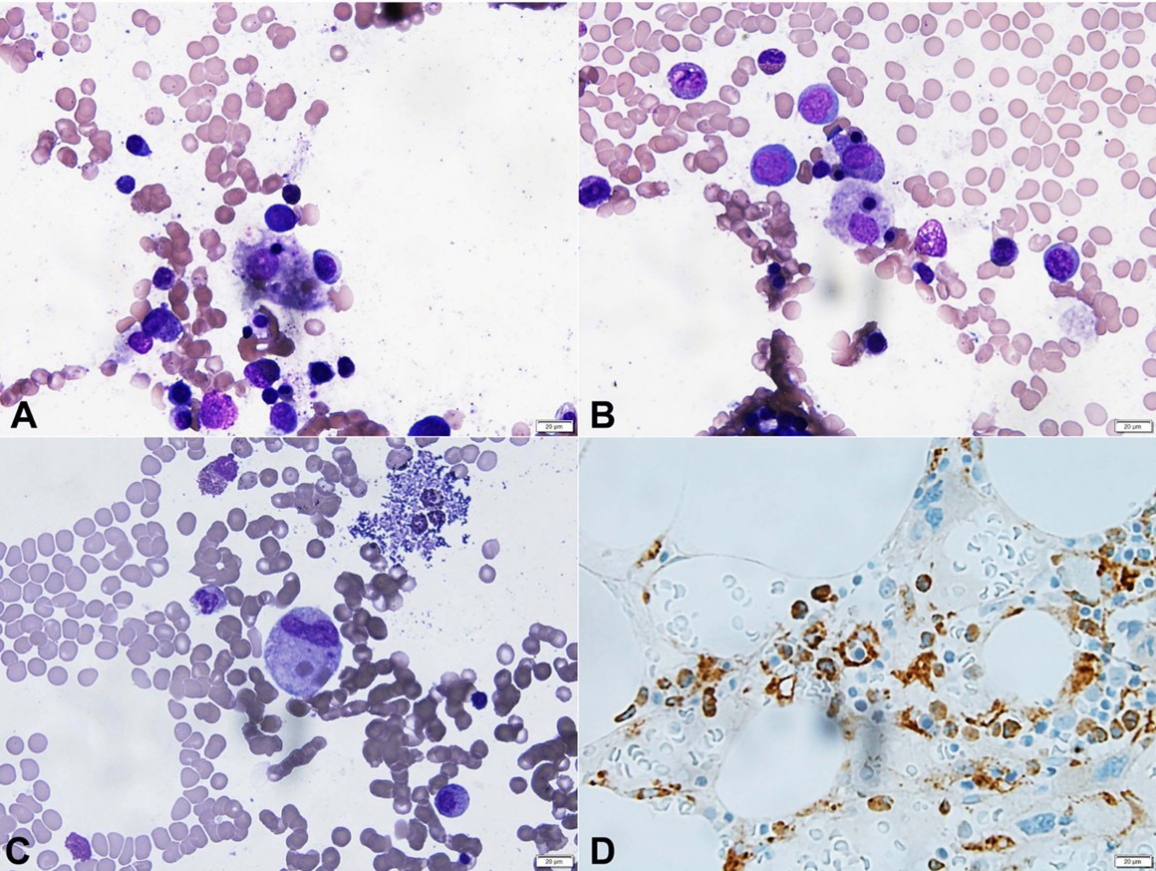
**A, B and C –** Photomicrographs of bone marrow aspirate demonstrating hemophagocytosis (Wright stain; 600x); **D –** Photomicrograph of CD68 immunostain highlighting histiocytes (600x).

On hospital day six, her liver and renal injuries progressed (AST 10,372 U/L, ALT 846 U/L, ALP 157 U/L, total bilirubin 1.9 mg/dL (RV 0.2 – 1.19 mg/dL), INR 2.2; creatinine 6.55 mg/dL, BUN 45.09 mg/dL) and continuous renal replacement therapy (CRRT) was initiated. Serologies were positive for HSV-1 (DNA was detected by PCR) and acyclovir treatment (dosed for CRRT) was initiated. Bone marrow biopsy with aspirate were significant for hemophagocytosis (Figure 1A-D). Serum soluble IL-2 receptor levels were elevated (24,953 U/mL). Etoposide therapy was then initiated.

The patient’s clinical condition continued to deteriorate requiring vasopressors and mechanical ventilatory support. Her metabolic acidosis worsened and she developed seizure-like activity. She was transitioned to comfort care and passed away on hospital day seven.

## AUTOPSY FINDINGS

**Figure 2:**
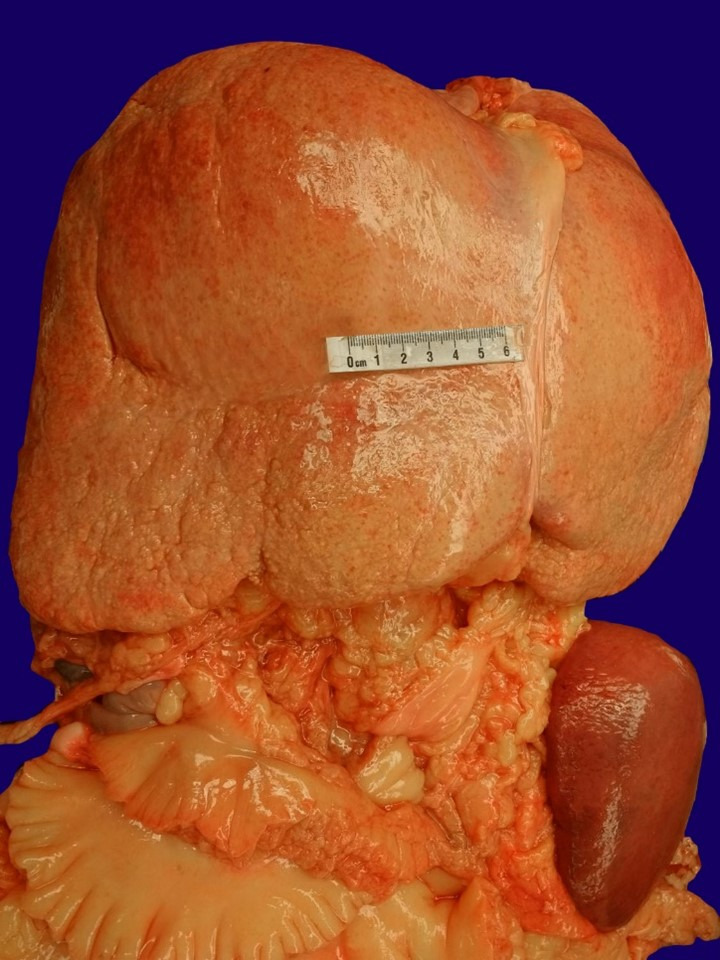
Photograph of organ block at postmortem examination highlighting the hepatosplenomegaly.

Postmortem examination showed findings consistent with a history of chronic steroid use including cushingoid features and striae over the chest and abdomen. Additional macroscopic findings included cardiomegaly and moderate atherosclerosis of the left anterior descending coronary artery, hepatosplenomegaly with the enlarged liver weighing 4,140 g (RV 1500-1800 g) and the spleen weighing 570 g (RV 155 g) (Figure 2).

**Figure 3:**
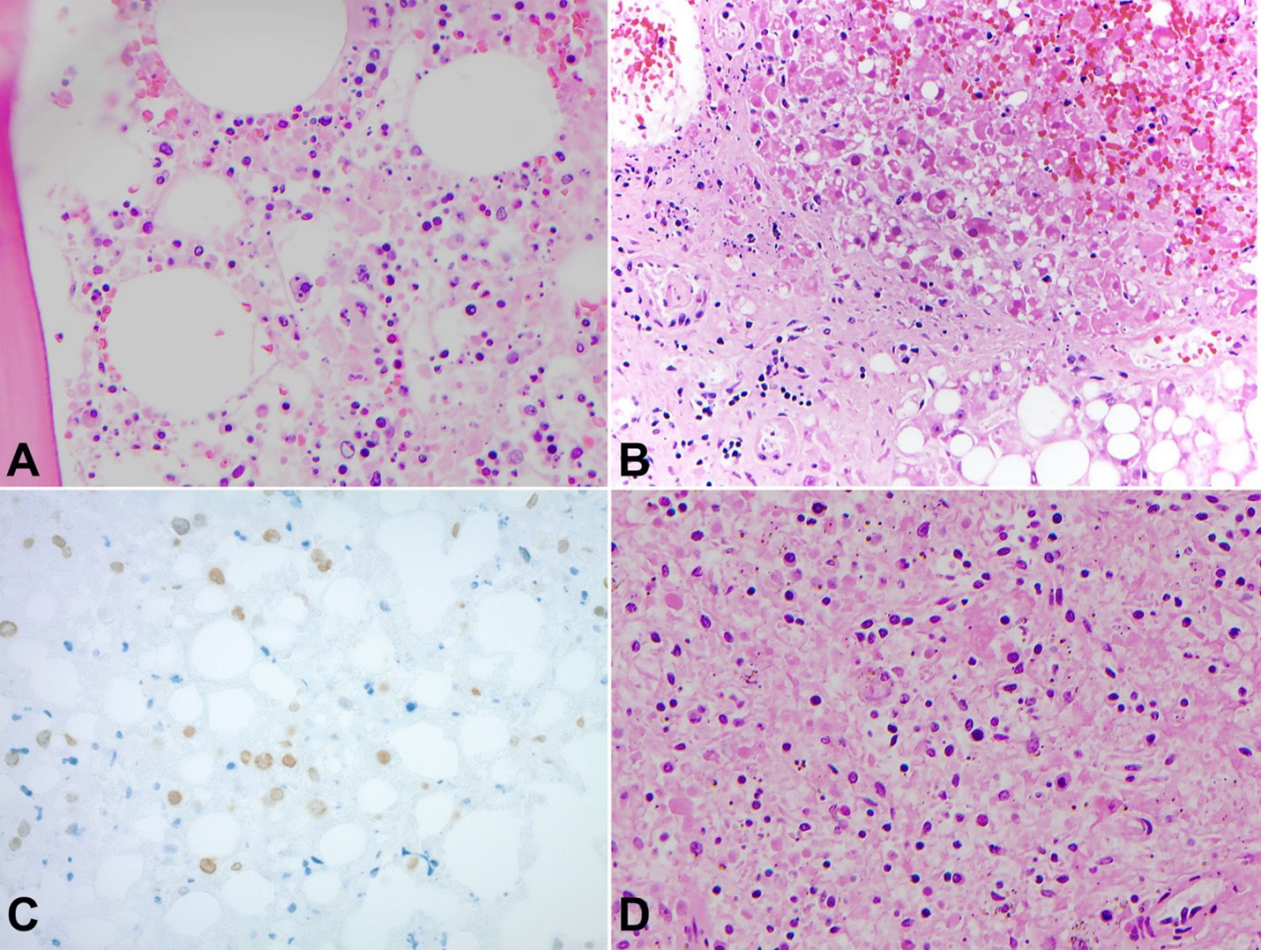
**A –** Photomicrograph of decalcified vertebral bone marrow sampled at autopsy demonstrating hemophagocytosis (H&E, 400x); **B –** Photomicrograph of liver sampled at autopsy with viral cytopathic changes, nuclear debris and macrovesicular steatosis (H&E, 200x); **C –** Photomicrograph of liver sampled at autopsy with macrovesicular steatosis and HSV1-2 immunopositive hepatocyte nuclei (200x); **D –** Photomicrograph of spleen sampled at autopsy demonstrating significant autolysis with occasional enlarged phagocytic cells present (center) (H&E, 400x).

Histologic findings were significant for hemophagocytosis in the bone marrow (Figure 3A) and for macrovesicular steatosis with bridging fibrosis of the liver, which additionally demonstrated viral cytopathic changes and nuclear debris (Figure 3B). Immunohistochemical stain for HSV-1/2 was positive within the liver (Figure 3C). The spleen demonstrated extensive autolytic changes, but occasional large phagocytic cells are appreciable (Figure 3D).

Aside from the presence of a patent ventriculoperitoneal shunt, examination of the head and central nervous system showed no other significant gross or histologic findings. The kidneys showed acute tubular injury and the lungs were grossly and microscopically unremarkable; thus, there was no evidence of GPA activity.

## DISCUSSION

This case illustrates a fatal outcome of hemophagocytic lymphohistiocytosis driven by viral infection in the setting of immunosuppression. HLH is a rare, aggressive hyperinflammatory syndrome in which an inciting event triggers massive uninhibited activation of T lymphocytes and macrophages.^1^ HLH-2004 diagnostic criteria established by the Histiocyte Society requires five of the following eight diagnostic criteria: (i) fever; (ii) hepatosplenomegaly; (iii) cytopenias (affecting two or more lineages in the peripheral blood); (iv) hypertriglyceridemia and/or hypofibrinogenemia; (v) hyperferritinemia; (vi) hemophagocytosis in bone marrow, spleen, or lymph nodes; (vii) low or absent natural killer (NK)-cell activity; and (viii) elevated soluble CD25 (interleukin [IL]-2 receptor).^2^ Early diagnosis and treatment of HLH is crucial for survival. However, it is necessary to exclude more common, clinically similar entities such as sepsis and liver failure.^1^^,^^3^ Distinguishing HLH from sepsis is difficult in critically ill patients on chronic immunosuppressive therapy with transaminitis and evidence of systemic inflammatory response syndrome (SIRS). While the presence of fever, hyperferritinemia, hepatosplenomegaly, cytopenia, and rapidly progressing multiorgan system failure were clinically suspicious for HLH, these findings also overlap with disseminated HSV infection.^4^ Elevated soluble IL-2 receptor levels and bone marrow biopsy showing hemophagocytosis, although also unspecific, filled the diagnostic criteria for HLH.

HLH has a wide variety of triggers including infection, malignancy, autoimmune disease, chronic diseases, and drugs. Multiple underlying triggers are identified in about one third of adults with HLH.^3^ In this case of a young woman with a history of ANCA-associated vasculitis on immunosuppressive therapy with multiple comorbidities, macrophage activation syndrome (MAS) was also a part of the differential. MAS is a subset of HLH triggered by autoimmune disease. MAS most commonly occurs in patients with adult-onset Still’s disease and systemic juvenile idiopathic arthritis. Although rare, cases of HLH in patients with rheumatoid arthritis, SLE, and systemic vasculitis have been reported.^5^ In our patient, the premortem rheumatology workup and postmortem examination showed no evidence of GPA activity.

An extensive infectious workup during the patient’s hospital stay was significant only for HSV-1 detected on serum PCR. Blood and lung cultures collected at autopsy were negative. Viral infection is the most common trigger of HLH in adults. Acute viral infections can act as a trigger of previously called primary and secondary HLH. A review of 2,197 cases found that the Herpesviradae family was responsible for 62% (n = 473) of virus-associated HLH cases in adults. Of these, EBV was the most common (43%, n = 330), followed by CMV (9%, n = 69) and other herpesviruses (10%, n = 74). Cases of HSV, HHV-6, HHV-8, VZV, and parvovirus 19 were also reported.^3^

HSV-1 typically triggers HLH in neonates but rarely in adults. To our knowledge, six cases of HSV-1 induced HLH have been previously reported in adults, only two of which were fatal.^6^^-^11 Drori et al.^6^ describe the case of a 50-year-old man with a past medical history significant for diabetes mellitus who initially presented as an outpatient with fever, leukopenia, and dyspnea. Despite the antibiotic treatment and a negative infectious workup, his condition deteriorated over the next week and he was admitted to the hospital with fever, hyperferritinemia, and acute liver and kidney failure. An abdominal ultrasound showed splenomegaly and the bone marrow biopsy showed no evidence of hemophagocytosis. A solitary palpable axillary lymph node was not biopsied. Etoposide treatment was scheduled due to clinical suspicion for HLH. However, the patient passed away 12 hours after admission.

Honsig et al.^7^ discuss a similar case of a 21-year-old of unspecified gender with no significant past medical history who presented with fever, leukopenia and thrombocytopenia, mild transaminitis, hyperferritinemia, and urinary tract infection. An initial infectious workup was positive for EBV. Splenomegaly and hepatic lesions were detected by MRI. The patient rapidly developed acute liver and kidney failure and was transferred to a tertiary care center where genital lesions consistent with HSV infection were noted. Despite acyclovir therapy, the patient passed away on day six of hospitalization. The cause of death was attributed to HLH induced by EBV and HSV-1 coinfection.

In both cases, postmortem studies were limited and included liver biopsy and blood draws. The liver biopsies showed areas of focal necrosis and ground-glass eosinophilic inclusions, which were positive for HSV-1 by immunohistochemistry. The findings were confirmed by PCR of the hepatic tissue and postmortem serum analysis in both cases included positivity for HSV-1 by PCR and elevated soluble IL-2 receptor levels.^6^^,^^7^

Cusini et al.^8^ describe the case of a 57-year-old man with a history of GPA requiring dialysis who presented with fevers and computed tomography (CT) findings consistent with acute cholecystitis. Two weeks following cholecystectomy, the patient continued to be febrile and clinically deteriorated requiring mechanical ventilation. Laboratory values were significant for hyperferritinemia, pancytopenia, and elevated soluble IL-2 receptor. A bone marrow biopsy showed hemophagocytosis. An infectious workup detected HSV-1 in both peripheral blood and cerebral spinal fluid. The patient was treated with acyclovir, high dose steroids, and etoposide. He ultimately recovered and was discharged after thirteen weeks.

The remaining reported cases of isolated HSV-1 induced HLH occurred in three immunocompetent individuals ranging in age from 52-69 years old with no significant past medical histories. Two of these patients recovered after receiving acyclovir, intravenous immunoglobulin, and etoposide or acyclovir alone and the third patient went on to require liver transplantation following acyclovir therapy.^9^^-^11

## CONCLUSION

HLH is a rare, aggressive hyperinflammatory syndrome with a wide variety of triggers. Early diagnosis and treatment are imperative for optimal patient outcome. While the Herpesviridae family is responsible for the majority of cases triggered by viral infection, HSV-1 induced HLH is extremely uncommon in adults. This report illustrates the diagnostic challenges of diagnosing and determining the etiology of HLH in a medically complex patient.
